# Manifold Routes to a Nucleus

**DOI:** 10.3389/fmicb.2018.02604

**Published:** 2018-10-26

**Authors:** Heather L. Hendrickson, Anthony M. Poole

**Affiliations:** ^1^Institute of Natural and Mathematical Sciences, Massey University, Auckland, New Zealand; ^2^Bioinformatics Institute, The University of Auckland, Auckland, New Zealand; ^3^Te Ao Mârama/Centre for Fundamental Inquiry, The University of Auckland, Auckland, New Zealand; ^4^School of Biological Sciences, The University of Auckland, Auckland, New Zealand

**Keywords:** nucleus, compartmentation, planctomycetes, jumbophage, Asgard, RNA avoidance

## Abstract

It is widely assumed that there is a clear distinction between eukaryotes, with cell nuclei, and prokaryotes, which lack nuclei. This suggests the evolution of nuclear compartmentation is a singular event. However, emerging knowledge of the diversity of bacterial internal cell structures suggests the picture may not be as black-and-white as previously thought. For instance, some members of the bacterial PVC superphylum appear to have nucleus-like compartmentation, where transcription and translation are physically separated, and some jumbophages have recently been shown to create nucleus-like structures within their Pseudomonad hosts. Moreover, there is also tantalizing metagenomic identification of new Archaea that carry homologs of genes associated with internal cell membrane structure in eukaryotes. All these cases invite comparison with eukaryote cell biology. While the bacterial cases of genetic compartmentation are likely convergent, and thus viewed by many as not germane to the question of eukaryote origins, we argue here that, in addressing the broader question of the evolution of compartmentation, other instances are at least as important: they provide us with a point of comparison which is critical for a more general understanding of both the conditions favoring the emergence of intracellular compartmentation of DNA and the evolutionary consequences of such cellular architecture. Finally, we consider three classes of explanation for the emergence of compartmentation: physical protection, crosstalk avoidance and nonadaptive origins.

## The Conundrum of the Eukaryote Nucleus

**FIGURE 1:**
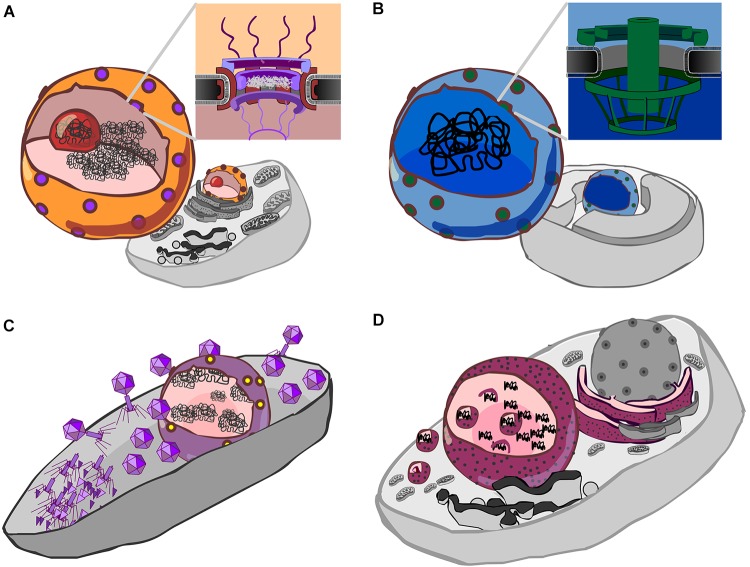
Forms of genetic compartmentation. **(A)** Cartoon depiction of a eukaryote cell and nucleus (orange) with nuclear pores (purple), nucleolus (red) and DNA (black). Inset: a schematic cross-section of the nuclear pore complex with a central plug, cytoplasmic fibrils above, and basket structure below the plane of the membrane. The inner and outer membranes of the nuclear envelope are continuous. **(B)** Cartoon depiction of a *Gemmata obscuriglobus* cell showing internal membranes (Fuerst and Sagulenko, 2011), a proposed nucleus-like structure (blue) which contains DNA, and pores (green). Inset: a schematic of the nuclear pore-like structure reported by Sagulenko et al. (2017). **(C)** Cartoon detailing the Jumbo phage proteinaceous nucleus-like structure (purple) containing phage DNA (black), after Chaikeeratisak et al. (2017b). Protein synthesis and viral assembly occurs outside this structure, whereas DNA replication and transcription occur within. This suggests the presence of a mechanism for both export of RNA and DNA, depicted here as a single complex by yellow dots. **(D)** Cartoon detailing co-option of the endoplasmic reticulum by Mimivirus, a Nucleocytpplasmic Large DNA virus (NCLDV). Here, the rough endoplasmic reticulum (maroon with black speckles) is recruited to form an encapsulating virus factory (Mutsafi et al., 2013). A complex series of events then leads to formation of viral capsids which are then encapsulated by protein-coated membranes (Kuznetsov et al., 2013) (depicted by small magenta spheres).

The eukaryote nucleus (Figure 1A) is one of the most remarkable structures in biology. It is home to the major part of the genetic material in eukaryotic cells, and is conserved across all eukaryotes, which share a core set of genes for the nuclear pore complex (Mans et al., 2004; Bapteste et al., 2005; Neumann et al., 2010) and nucleocytoplasmic transport (Koumandou et al., 2013). Much speculation on the origin of the eukaryote nucleus has been published, and models fall into two broad classes: endosymbiotic and autogenous (Martin et al., 2001). In endosymbiotic models, the nucleus is proposed to have evolved from a once free-living cell or from a virus (Bell, 2001; Takemura, 2001; Forterre and Prangishvili, 2009; Forterre and Gaia, 2016), whereas in most autogenous models, the nucleus evolved through internal changes that led to compartmentalization of the genetic material (Devos et al., 2004). The question of eukaryote origins has been given new fuel via the recent metagenomic identification of a new group of Archaea, called Asgard (Spang et al., 2015; Zaremba-Niedzwiedzka et al., 2017), which encode putative homologs to eukaryotic nucleocytoplasmic transport genes (Klinger et al., 2016). If the function of these archaeal genes can be established and related to cell ultrastructure, it will assuredly improve our understanding the evolution of eukaryotes (Dey et al., 2016), given a shared evolutionary history with the Archaea.

However, it is difficult to fully untangle the evolution of singular events (De Duve, 2005): did nuclear compartments evolve only once, thus requiring some special evolutionary explanation (Lane, 2011), or can they be understood with reference to known processes (Poole and Penny, 2007; Booth and Doolittle, 2015)? Researchers trying to understand the origins of life know this dilemma well: all cellular life on earth derives from a single origin. While it is possible that there were multiple origins of life on Earth (Davies and Lineweaver, 2005), there is no evidence that extant cells derive from independent origins (Penny et al., 2003). Thus, reconstructing the origin and evolution of life on earth provides us with the history of a specific instance. To understand the broader process of how life originates, it would be helpful – perhaps essential – to be able to compare life on earth with life that derives from one or more independent origins (McKay, 2004). This would help us to understand whether there is only one way that life can originate, or if there are there many routes.

In this spirit, we consider here the broader question of the origins of genetic compartmentation, of which the origin of the eukaryote nucleus has widely been assumed to represent the single instance. In contrast to the origin of life, the origin of genetic compartmentation is no longer a singularity; multiple forms of genetic compartmentation have now been observed, including among bacteria. This enables us to pose a more general question, of which the origin of the eukaryote nucleus is a specific historical instance: what forces drive the compartmentalization of genetic information within cells?

## Nucleus-Like Compartmentation is Found Outside of Eukaryotes

Members of the bacterial “PVC” superphylum, which comprises the Planctomycetes, Verrucomicrobia and Chlamydiae, appear unusual among bacteria in that a number have internal membranes (Lee et al., 2009; Fuerst and Sagulenko, 2011). The most studied member of this group, the planctomycete *Gemmata obscuriglobus*, possesses a nucleus-like compartment that contains DNA (Fuerst, 2005; Sagulenko et al., 2014), and exhibits physical separation of transcription and translation (Gottshall et al., 2014). Moreover, this nucleus-like compartment possesses membrane-spanning pores that are about a third the diameter of the eukaryote nuclear pore complex, and bear unmistakeable resemblance to eukaryote nuclear pores, both in overall architecture (Figure 1B), and in regard to the kinds of protein domains (beta-propellers, alpha solenoids) identified within its protein constituents (Sagulenko et al., 2017). Interestingly, it does appear that some ribosomes are found in the same compartment as the genetic material (Fuerst and Sagulenko, 2011), so the extent to which translation and transcription are separated by this compartmental barrier is unclear. While some have speculated that the nucleus-like structure in planctomycetes such as *G. obscuriglobus* could belie an ancient origin for nuclear compartmentation (Fuerst and Sagulenko, 2012; Staley and Fuerst, 2017), it seems more likely to be a stunning case of evolutionary convergence (Sagulenko et al., 2017), though it is worth noting that the exact nature of this compartmentation is a matter of ongoing debate (Acehan et al., 2014; Sagulenko et al., 2014, 2017; Boedeker et al., 2017).

Of importance here is whether the probable convergence of the intracellular compartmentation of *Gemmata* renders such compartmentation irrelevant to evolution of the eukaryote nucleus (McInerney et al., 2011). From an historical perspective, this is true: an understanding of the specific evolutionary history of bird wings does not shed light on the evolutionary history of flight in bats. But consider for a moment the value of a separate origin: comparison of the two kinds of flight reveals separate evolutionary solutions to the same problem. If planctomycete nuclear-like compartmentation does hold up to closer scrutiny, then it seems difficult to explain it via existing models for the origin of the nucleus. For instance, the suggestion that intron invasion necessitated a separation of splicing and translation (Lopez-Garcia and Moreira, 2006; Martin and Koonin, 2006) does not explain the cellular organization of *Gemmata*, not least because Planctomycetes lack an intron-exon gene organization and a mechanism for splicing. Thus, while Asgard lineages promise to shed light on the intermediate steps in eukaryogenesis (Eme et al., 2017; Fournier and Poole, 2018), the independent emergence in Planctomycetes of a nucleus-like compartment with separated transcription and translation may instead shed light on the range of mechanisms by which this type of architecture may evolve.

## Jumbophage Form Proteinaceous “Nucleus-Like” Compartments

Another fascinating case of genetic compartmentation in bacteria has recently been discovered. During infection of *Pseudomonas chlororaphis* by jumbophage 201_Φ_2-1, a genetic compartment is formed (Chaikeeratisak et al., 2017b). Jumbophages have very large genomes, the largest of which currently stands at 497.5 kb (Bacillus phage G; GenBank accession: JN638751) (Hendrix, 2009; Yuan and Gao, 2017). Following host infection, jumbophage 201_Φ_2-1 forms a dynamic protein-based compartment within the body of the bacterial cell (Figure 1C). Within this structure, phage DNA is replicated and transcribed whilst phage mRNA is transported outside of the compartment where it is translated, enabling the construction of phage particles (Chaikeeratisak et al., 2017b). In this regard, this protein-based partitioning is “nucleus-like”—transcription is separated from translation—though it is clearly not homologous to the eukaryote nucleus. This may be a more widespread phenomenon; phages _Φ_PA3 and _Φ_KZ, that infect *P. aeruginosa*, also generate this kind of nucleus-like structure. In all three cases, infection involves PhuZ, a tubulin homolog that forms a spindle that serves to position the nucleus, much like tubulin in eukaryotes, and gp105, a nuclear shell protein (Chaikeeratisak et al., 2017a,b). Homologs of both are detectable in 5 of the 8 complete *Pseudomonas* jumbophage in Genbank, plus in two recently sequenced jumbophages from New Zealand (HH, unpublished observations), suggesting this may be a more widespread process.

Given that the origin of the nucleus remains an unsolved problem in biology, the formation of a proteinaceous compartment during jumbophage infection invites parallels to viral-origin models for the eukaryote nucleus. In this class of model, cooption of internal membranes by infecting viruses drove stable formation of a nuclear compartment (Bell, 2001, 2009; Takemura, 2001; Forterre and Gaia, 2016). The comparison that is frequently made is with members of the NucleoCytoplasmic Large DNA Viruses (NCLDV) group, which recruit internal host membranes (Kuznetsov et al., 2013; Mutsafi et al., 2013) to create an intracellular compartment (Figure 1D). By contrast, the *de novo* formation of the jumbophage proteinaceous compartment indicates that compartments can be directly generated by virus infection. As this process happens in unrelated, genomically large, bacterial and eukaryote viruses, a more general question is this: might there a benefit to compartmentalization of the genetic material of large viruses? And what of compartmentalization more generally?

## What Drives the Compartmentation of Genetic Information?

Indeed, the evolutionarily independent forms of compartmentation in eukaryotes, the PVC superphylum, NCLDV viral factories and jumbophage nuclei enable us to consider more generally what might drive compartmentation of genetic material. The open questions are: do any of these separate instances share a common driver or are they the result of unrelated evolutionary pressures? Is compartmentation neutral or non-adaptive? To begin to address these, we next consider three broad categories of explanation that might account for the emergence of compartmentation: physical protection, crosstalk avoidance and non-adaptive origins; we explain each in turn.

### Protection

#### Chemical Protection

**FIGURE 2:**
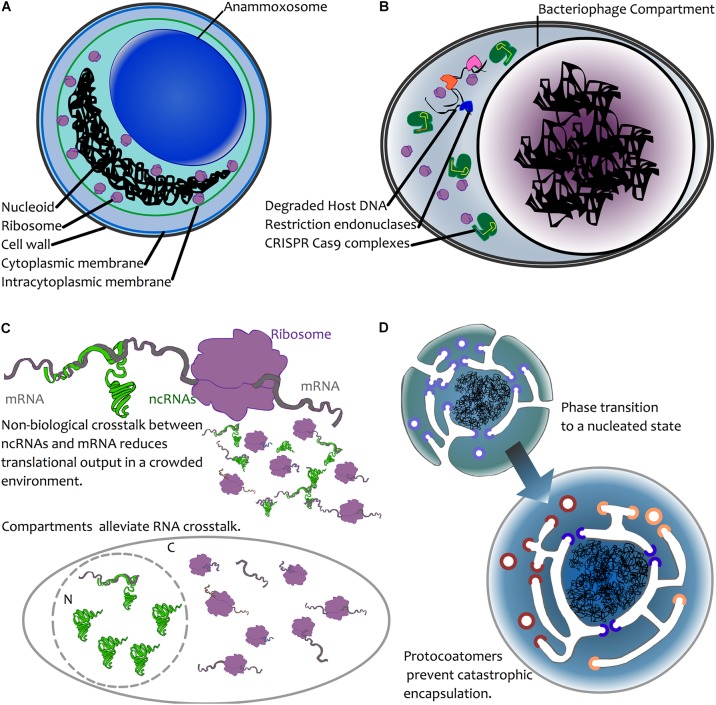
Possible mechanisms for compartmentalization of genetic material. **(A)** Chemical sequestration—as seen in annamox bacteria—where anaerobic ammonium oxidation is sequestered away from the rest of the cell in a dedicated compartment, the anammoxosome (van Niftrik, 2013). **(B)** Biological sequestration—the creation of dedicated viral compartments by large viruses (NCLDVs, Jumbophage) may prevent the action of host defenses or competition by other infectious agents by excluding these during biogenesis. For simplicity, an infected bacterial cell is depicted here, with CRISPR-Cas (green) and restriction endonuclease-based defense mechanisms excluded from the compartment. Restriction endonucleases are depicted in blue, pink orange. **(C)** Biological avoidance. Top: mRNA (grey) may interact with a ribosome (purple), leading to translation. In the case of ncRNA-mRNA interactions, these may occur through chance base-pairing, leading to reduced translational output. This is depicted by the smaller molecules, where some mRNAs interact with ribosomes, and some are trapped in unproductive interactions with ncRNAs (see text for details). In both prokaryotes and eukaryotes, avoidance of such crosstalk is a result of selection against interactions (Marco, 2015; Umu et al., 2016). Bottom: compartmentation is hypothesized to alleviate crosstalk interactions due to physical separation. The presence of ribosomes in the cytoplasm (C) and ncRNAs in the nucleus (N) reduces the opportunity for ncRNA-mRNA crosstalk to impact translation. **(D)** Biophysical nucleation. Top left: a protoeukaryotic cell possessing endomembranes and protocoatomers (violet) that enable membrane bending (Devos et al., 2004). Bottom right: following a biophysical phase transition (Braun, 2008), the DNA becomes spontaneously encapsulated. The presence of protocoatomers prevents “catastrophic” encapsulation by forming protopores following membrane fusion. Protopores later diversify into nucleoporins (blue) and protocoatomers into coatomers (red, orange) (Devos et al., 2004).

Keeping DNA physically separate from certain enzymatic reactions could act as a means of limiting chemical damage to the DNA. Under this model, damage would be prevented because the DNA is physically separated from damaging chemistries. It seems highly unlikely that the nuclear pores, which allow passive diffusion of small (30–60 kDa) molecules (Timney et al., 2016), or their smaller planctomycete analogues (Sagulenko et al., 2017) substantially reduce chemically induced DNA damage; in eukaryotes, mitochondria and chloroplasts are key sites of redox chemistry that may damage DNA, and this may be but one driver of gene relocation to the nucleus (Race et al., 1999). In the anammoxosome in planctomycetes (the site of anaerobic ammonia oxidation), specialized compartments contain the toxic intermediate of annamox chemistry (Sinninghe Damste et al., 2002) (Figure 2A), and this is much the same with eukaryotic peroxisomes (Gabaldon, 2010). Rather than sequestering DNA from potentially damaging chemistries, it seems the opposite occurs: it is the damaging chemistries that are compartmentalized. In lineages adapted to high levels of mutational damage, such as *Deinococcus radiodurans*, protection is via high ploidy (providing genome redundancy) and a resistance of the proteome to damage (without working repair proteins, there can be no DNA repair) (Daly et al., 2007; Slade et al., 2009; Krisko and Radman, 2013). It therefore seems that compartmentalisation of DNA is at best an infrequent solution to chemical or environmental sources of DNA damage.

#### Biological Protection

A more plausible argument for compartmentation of DNA may be as a means of protection from biological agents. In the case of viruses, a physical barrier may provide a powerful means by which to thwart host defenses. Many anti-phage systems have evolved and many of these rely on physical access to phage DNA in order to act, including CRISPR-Cas, restriction endonucleases and abortive infection systems (Labrie et al., 2010; Koonin et al., 2017). The discovery of the jumbophage “nucleus” prompted speculation that encapsulation of the phage DNA during infection provides an effective physical barrier for guarding the DNA from host defense systems (Figure 2B) (Chaikeeratisak et al., 2017b). In a recent article posted to BioR_x_iv, Mendoza et al. (2018) directly test this, and report that jumbophage _Φ_KZ is resistant to several CRISPR-Cas systems and type I restriction-modification systems, but is sensitive to CRISPR-Cas13a, which can target phage mRNA that must exit the shell for translation. Clearly, this is not a general strategy among viruses, but it might be the case that large viruses represent both a bigger genomic target and that they have enough genomic space to be able to carry larger suites of genes devoted to thwarting host defenses. In addition, their burst sizes are an order of magnitude lower than many smaller viruses, so physical protection may simply be less of an issue for viruses where infection yields many more virions. One might therefore expect physical barriers to be a mechanism common among large viruses. Certainly, this appears consistent with the lifecycle of NCLDVs, which co-opt internal membrane material during infection. That said, it may be difficult to tease apart protection and organization; while the large viruses of eukaryotes often form “viral factories” by coopting host membranes, this process appears to vary considerably (Novoa et al., 2005) and it is notable that the viral factory of Mimivirus appears not to be surrounded by a membrane (Suzan-Monti et al., 2007), though, having said that, it does appear that the capsid self-assembles on vesicles that derive from the nuclear membrane or rough endoplasmic reticulum (Kuznetsov et al., 2013).

More generally, nuclear envelopes in eukaryotes and planctomycetes could reduce opportunities for integration of foreign DNA, thus acting as a possible barrier to infection or invasion of genetic elements. At the level of phylum, available data are consistent with this interpretation; Jeong et al. (2016) recently calculated HGT indices (N_horizontal_/N_total_ genes) for major bacterial and archaeal phyla, and report that Planctomycetes, Verrucomicrobia and Chlamydiae all have HGT indices well below the global median, with the first two phyla being among the lowest in their analysis. This could be further tested by examining rates of HGT in lineages with and without nuclear compartmentation; the PVC superphylum could be a good test-bed for this as there are a range of cellular architectures across this group (Fuerst and Sagulenko, 2011). Nevertheless, eukaryote horizontal gene transfer rates are appreciable (Leger et al., 2018), it is unclear that gene transfer is selected against, and, relative to prokaryotes, eukaryotic nuclear genomes do not show evidence of an improved capacity to repel invasive genetic elements; genomic evidence suggests quite the opposite in fact (Werren, 2011). Accumulation of such elements appear instead to be linked to the mode of reproduction (spread of elements is most effective under sexual outcrossing) (Arkhipova and Meselson, 2000) and the capacity to purge slightly deleterious mutations (Lynch et al., 2011). That said, the barrier provided by the nucleus does act to slow the invasion of retroelements that must enter the nucleus to replicate. Several yeast nucleoporin genes are under positive selection, and genetic changes to these impact the propensity of Ty retrotransposons to replicate in their host (Rowley et al., 2018). This indicates that the nuclear envelope can directly impact replicative spread of genetic elements in the nucleus. It is thus plausible that the nuclear envelope first evolved to slow the spread of transposable elements following the evolution of meiosis.

### Crosstalk Avoidance

Crosstalk, i.e., unintended interaction between molecules, has been considered as a possible mechanism that could drive nuclear compartmentation (Figure 2C). One model for this is avoidance of ribosomal readthrough into introns (Lopez-Garcia and Moreira, 2006; Martin and Koonin, 2006). Under this model, crosstalk is a temporal problem, where interactions must occur in a specific order; the physical separation of transcription and translation may thus provide a temporal means for splicing to complete before translation begins, therefore preventing the formation of aberrant proteins via translational readthrough into unspliced introns, which may have toxic effects. The model is plausible, but, from a genetic perspective, it would work only under conditions of sexual reproduction, since intron invasion is limited under asexual reproduction (Poole, 2006). This model is relevant to eukaryote nuclear origins, but may not apply to other cases: the separation of transcription and translation (Gottshall et al., 2014) and presence of a nuclear envelope-like structure in *Gemmata* (Sagulenko et al., 2017) cannot be explained under this model, not least because *Gemmata* does not possess introns.

Avoidance of crosstalk may nevertheless be relevant to compartmentation. A recent study (Umu et al., 2016) demonstrated that there is selection for reduced crosstalk between highly expressed mRNAs and ncRNAs in a wide array of bacteria and archaea. Failure to avoid interaction with ncRNAs leads to reduced levels of protein expression, and hence it appears that such interactions have been selected against. For larger genomes with larger numbers of genes, it may be more challenging to avoid crosstalk interactions between ncRNAs and mRNAs. Moreover, where population sizes are also reduced (as expected in the origin of eukaryotes (Lynch, 2007)), this may reduce the capacity for crosstalk interactions to be selected against. Spatial separation of ncRNAs and mRNAs via compartmentation may thus alleviate crosstalk in eukaryotes, where weaker selection and larger numbers of transcriptional outputs would yield more opportunities for crosstalk. Indeed, in eukaryotes, where there is extensive RNA-based regulation, it is noteworthy that mRNA maturation (nucleus) and miRNA maturation (cytoplasm) are physically separated. Also suggestive of crosstalk avoidance, eukaryotic microRNAs are synthesized in a stem-loop precursor form (pre-miRNA) that, through internal base pairing, precludes crosstalk. Indeed, there is evidence for crosstalk avoidance in miRNAs during early embryonic development in *Drosophila* (Marco, 2015). Given that, at the very early stages of *Drosophila* development, multiple nuclear divisions occur in a syncytium without formation of cell membranes (Lawrence, 1992), the opportunities for crosstalk may be greater. For RNAi in general, it is only following export from the nucleus that the miRNA is processed by Dicer, and the interaction between mature miRNA and target mRNA occurs within the RNA-induced silencing complex (RISC) (Wilson and Doudna, 2013), which may again reduce the opportunities for cross-talk. Avoiding crosstalk may thus have been a factor in the evolution of the eukaryote nucleus, and possibly in bacteria with large genomes such as *G. obscuriglobus*.

In the case of jumbophage, avoidance might also be a driver of compartmentation, though for different reasons: it is tempting to consider that compartmentation might contribute to infection success by competitive exclusion of resident prophage.

### Non-adaptive

A final possibility is that compartmentation, rather than being the result of natural selection, can in some cases non-adaptively emerge as a thermodynamic consequence of the amount of DNA present in a cell (Braun, 2008). In this model, colloidal phase separation in a crowded environment may occur if the genome is sufficiently large that the DNA nucleoid acts as a physical nucleation site (Figure 2D). Based on calculations that depend on intracellular volume and the number of expressed macromolecules, Braun (2008) estimated that formation of nucleoids may spontaneously occur for genomes that are ∼10 Mbp in length. In short, large genomes render the nascent growth of a compartment thermodynamically favorable. As appealing as this model is, it raises many questions. There are a few species of bacteria that have genomes ∼10 Mbp, and, notably, this does include *G. obscuriglobus* (∼9.2 Mbp). However, there are many bacteria that have very high genome copy number. For instance, the bacterium *Azotobacter vinlandii* has a chromosomal copy number exceeding 100 (Bendich and Drlica, 2000). This would push the total base-pair count to over an order of magnitude greater than Braun’s calculations. Under this model, polyploid cells would need to be extremely large to avoid nucleation! Details aside, this model does not directly explain the formation of a membrane around the nucleoid, and lipid encapsulation may be fatal if transport is not already developed. However, it bears considering here as it raises the possibility that, rather than there being some direct advantage to compartmentalization, this might occur simply by biophysical processes. We should be wary of taking it as given that there was a strong selective advantage to nuclear compartmentation; it may instead have been non-lethal. Perhaps early coatomers (Devos et al., 2004) simply prevented complete encapsulation of DNA by invaginated membrane (Figure 2D), and, prior to the advent of FG-repeat proteins, larger molecules were afforded free movement between protonucleus and cytoplasm through the resulting proto-pores.

## Future Perspectives

The origins DNA compartmentation remains a difficult problem. However, the observation that this is not restricted to the eukaryote nucleus enables us to move away from the temptation of requiring some special explanation for eukaryote nuclear origins, to more mechanistic explanations, be they through neutral or selective processes. That there appear to be manifold routes to nuclear compartmentation is exciting not because different instances may be evolutionarily related, but rather because multiple instances may help us shed light on whether compartmentation of DNA is advantageous or accidental. In the context of the latter possibility, it is important to bear in mind that an accidental compartment might well turn out to be advantageous at some future date, without being of immediate short-term value; the key question for such a model is whether it is non-lethal in the short term. With ever-improving tools for synthetic biology and experimental evolution, it may soon be possible to start addressing these questions in the lab.

## Author Contributions

Both authors conceived and wrote the paper together.

## Conflict of Interest Statement

The authors declare that the research was conducted in the absence of any commercial or financial relationships that could be construed as a potential conflict of interest.
